# Protection of IFNAR (−/−) Mice against Bluetongue Virus Serotype 8, by Heterologous (DNA/rMVA) and Homologous (rMVA/rMVA) Vaccination, Expressing Outer-Capsid Protein VP2

**DOI:** 10.1371/journal.pone.0060574

**Published:** 2013-04-12

**Authors:** Tamara Kusay Jabbar, Eva Calvo-Pinilla, Francisco Mateos, Simon Gubbins, Abdelghani Bin-Tarif, Katarzyna Bachanek-Bankowska, Oya Alpar, Javier Ortego, Haru-Hisa Takamatsu, Peter Paul Clement Mertens, Javier Castillo-Olivares

**Affiliations:** 1 The Pirbright Institute, Pirbright, Woking, Surrey, United Kingdom; 2 Centro en Investigación y Sanidad Animal, Valdeolmos, Madrid, Spain; 3 Centre for Drug Delivery Research, London School of Pharmacy, London, United Kingdom; Institut National de la Santé et de la Recherche Médicale, France

## Abstract

The protective efficacy of recombinant vaccines expressing serotype 8 bluetongue virus (BTV-8) capsid proteins was tested in a mouse model. The recombinant vaccines comprised plasmid DNA or Modified Vaccinia Ankara viruses encoding BTV VP2, VP5 or VP7 proteins. These constructs were administered alone or in combination using either a homologous prime boost vaccination regime (rMVA/rMVA) or a heterologous vaccination regime (DNA/rMVA). The DNA/rMVA or rMVA/rMVA prime-boost were administered at a three week interval and all of the animals that received VP2 generated neutralising antibodies. The vaccinated and non-vaccinated-control mice were subsequently challenged with a lethal dose of BTV-8. Mice vaccinated with VP7 alone were not protected. However, mice vaccinated with DNA/rMVA or rMVA/rMVA expressing VP2, VP5 and VP7 or VP2 alone were all protected.

## Introduction

Bluetongue virus (BTV) is an arthropod borne, non-enveloped virus of the genus *Orbivirus*, within the family *Reoviridae*. It is transmitted by biting midges of the genus *Culicoides* and infects ruminants causing severe haemorrhagic ‘bluetongue’ disease (BT) particularly in sheep and some species of deer [1], [2]. The BTV genome is composed of ten linear segments of dsRNA encoding seven structural and four distinct non-structural virus proteins (VP1–VP7 and NS1–NS4 respectively) [3], [4]. The genome segments are packaged within a three-layered icosahedral protein capsid [5], [6], [7], [8], [9]. The BTV outer-capsid layer is composed of VP2 and VP5 proteins, encoded by genome segments 2 and 6 (Seg-2 and Seg-6) respectively. The outer-core layer is formed by VP7 protein, encoded by Seg-7, while the inner-most sub-core shell is formed of VP3 protein, encoded by Seg-3 [3], [7].

VP2 is the most variable of the BTV proteins and is a major protective antigen. The specificity of its interactions with neutralising antibodies determines the identity of the 26 known BTV serotypes [6], [10], [11], [12]. Consequently, differences in the amino acid sequence of VP2 show a close correlation with virus serotype [10]. However, there are also differences within each serotype that reflect the geographic origin (topotype) of the virus isolate [11], [12].

Although the smaller BTV outer-capsid protein VP5 is also highly variable, its sequence only shows a partial correlation with virus serotype and VP5 by itself does not appear to raise neutralising antibodies [6], [13]. However, although studies of BTV neutralisation-escape mutants mostly showed changes in VP2, such changes were occasionally also observed in VP5 [14]. Studies of reassortant progeny viruses, derived from parental strains belonging to two different BTV serotypes, suggest that interactions between VP2 and VP5 can affect the serological properties of the virus, possibly by VP5 influencing the conformation of VP2 [15], [16]. BTV outer-core protein VP7 does not appear to be exposed on the surface of intact bluetongue virus-particles [17], although it can mediate both cell attachment and penetration by BTV core-particles during the initial stages of infection of insect cells or adult vector insects [18]. Antibodies to VP7 can also bind to and neutralise core particles, but do not reduce the infectivity of the intact virus [17].

Since 1998, BT outbreaks have spread across the entire Mediterranean region, caused by BTV serotypes 1, 2, 4, 6, 8, 9, 11, 16 and 25, in some cases involving more than one strain or ‘topotype’ of the same serotype [19], [20]. The first BT outbreak ever recorded in northern Europe, started during 2006, caused by a ‘western’ strain of BTV-8 from sub-Saharan Africa [11]. The outbreak was first reported in the Maastricht region of the Netherlands, although it may have started earlier the same year in Belgium [21]. From this initial introduction, BTV-8 spread across most of Europe, killing many thousands of animals and causing massive economic losses (European Commission. Restriction zones of bluetongue in Europe as of December 19, 2007, cited 2007 December 27, Available from http://ec.europa.eu/food/animal/diseases/controlmeasures/bluetongue_en.htm). The virus arrived in the UK during August 2007, transmitted by wind-borne infected midges from the outbreak regions on the European mainland [22]. Although initial control measures, relied primarily on restriction of animal movements from the BTV-8 infected areas, the use of an inactivated vaccine in the UK during early 2008 prevented the re-emergence of the disease. Subsequent vaccination campaigns in other northern European countries (France, Belgium, the Netherlands and Germany), together with widespread natural seroconversion (post-infection), resulted in rapid eradication of both BTV-8 and BTV-1 from the region.

Although inactivated BTV vaccines were effective in northern Europe, some concerns still exist over the reliability of inactivation for each vaccine batch [23]. They are also monospecific, offering little protection against subsequent infections by heterologous BTV serotypes and it is uncertain how long the protective and neutralising antibodies responses that are generated will last in a vaccinated animal.

Although live attenuated vaccines are also available for multiple BTV serotypes, and appear to be highly effective in endemic regions for protection of individual susceptible animals against clinical signs of the disease, they can themselves cause severe disease in naïve sheep from northern Europe [24]. They also pose a further risk of genome segment re-assortment between vaccine and field strains, potentially leading to the emergence of progeny strains with novel biological characteristics.

Since both the inactivated and live attenuated BTV vaccines generate antibodies to all of the virus proteins, it has not been possible to develop a reliable serological assay to ‘distinguish infected from vaccinated animals’ (DIVA assays). In the face of a widespread vaccination campaign (as seen in Northern Europe) this invalidates the use of serological screening methods for surveillance. Virus detection, characterisation and surveillance during the BTV outbreaks in northern Europe have therefore relied heavily on conventional or real-time RT-PCR assays [25], [26].

Recombinant vaccinia virus, recombinant canarypox virus and recombinant capripox virus have all been used successfully as gene delivery systems for BTV-vaccination [27], . The passage of Chorioallantois vaccinia virus Ankara (CVA) over 570 times in primary chick embryo fibroblast cells (CEF), led to attenuated replication and reduced virulence, generating a new virus known as the ‘Modified Vaccinia Ankara’ strain (MVA) [30], [31]. Recombinant MVA (rMVA) provides a promising vaccine-vector, which activates both branches of the immune system [32], with a well-established safety record and history of use as a vaccine for infectious diseases and malignancies [31], [33]. DNA-vaccines have also been used experimentally for BTV and other orbiviruses [28], [34], [35], [36], [37] and were recently used in a heterologous prime-boost vaccination strategy (DNA/rMVA), providing protection against BTV in a mouse model system [28], [37].

Published data suggest that a combination of the three major BTV proteins VP2, VP5 and VP7, gives better protection than VP2 and VP5, or VP2 alone [5], [28], [38]. Indeed, co-expression of the four major structural proteins of BTV (VP2, VP3, VP5 and VP7 expressed by recombinant baculovirus) results in their assembly, generating ‘virus like particles’ (VLP) that also raise both neutralising antibodies and a protective response in sheep [38]. In addition, *in vivo* expression of the three capsid proteins VP2, VP5 and VP7 from three separate rMVA following a DNA prime/MVA boost vaccination regime was required to confer protective immunity in a BTV mouse model [28]. However, other studies with BTV and other related orbiviruses indicate that complete protection can be achieved by sub-unit vaccines based on the VP2 protein alone [39], [40]. In addition, an MVA based vaccine expressing VP2 of African horse sickness virus (AHSV) Serotype 4 also provided complete protection in mice against homologous AHSV-4 challenge [41].

The recent development of a new murine model, based on adult IFNAR (−/−) mice, has facilitated the study of the immune response and the testing of new vaccines against BTV. IFNAR (−/−) mice are knockout mice lacking the β subunit of the interferon α/β receptor and can be a good animal model for BTV because they are able to support the in vivo growth of BTV and they also show viraemia and disease symptoms [42]. Commercial inactivated vaccines against BTV have been tested in these mice [42] and they show very similar results comparing with vaccinated sheep or cattle in terms of neutralising antibodies and viraemia [43]. Moreover, our previous results [28], [37], [41], [42] and other studies [44], [45], [46], [47] show that the IFNAR (−/−) infection model is useful for the definition of effective recombinant vaccine candidates against several viruses.

In this study we investigated the protective efficacy of rMVA and DNA vaccines expressing BTV-8 VP2 in the mouse model based on IFNAR (−/−) mice. On the other hand, we wanted to determine whether the presence of VP5 and VP7 was critical in inducing protective immunity of VP2 based vaccines and whether a DNA prime/rMVA boost vaccination regime was more efficient than an rMVA prime/rMVA boost vaccination approach. We report the use of a heterologous DNA/rMVA and a homologous rMVA/rMVA prime-boost vaccine strategy of either BTV-8 VP2 (as sole antigen), or a combination of VP2, VP5 and VP7, to protect IFNAR (−/−) mice against a lethal challenge with a virulent strain of BTV-8.

## Materials and Methods

### Cells and Virus

Primary chicken embryo fibroblasts (CEF), the continuous chicken embryo fibroblast cell line DF-1 and African green monkey kidney cells (Vero) were all obtained from the Microbiological Services and Central Service units of the Institute for Animal Health.

The BTV-8 (Belgium/06 isolate) virus used for challenge studies at Centro de Investigación en Sanidad Animal, INIA, Madrid (CISA), was originally isolated from a calf in Belgium in 2006. The BTV-8 strain ‘NET2006/07′ from the orbivirus reference collection (ORC) at IAH (http://www.reoviridae.org/dsRNA_virus_proteins/ReoID/BTV-isolates.htm) was used for virus neutralisation tests (VNT) in Vero cells.

### Generating DNA Vaccines

DNA vaccines were based on the pCI-neo Mammalian Expression Vector (Promega). To generate pCI-neo BTV-8 Seg-2, pCI-neo BTV-8 Seg-6 and pCI-neo BTV-8 Seg-7, we PCR amplified each BTV genome segment from plasmids pBRT7 BTV-8 Seg-2 NET2006/07, pBRT7 BTV-8 NET2006/07 Seg-6 and pBRT7 BTV-8 NET2006/07 Seg-7 using gene specific primers containing XbaI and NotI restriction sites (Table 1). The amplicons and recombinant pCI-neo were digested with XbaI and NotI and ligated using standard molecular cloning techniques and then sequenced using pCI-neo specific primers to identify the correct insert.

**Table 1:** Oligonucleotide primers.

Name of Primer	Primer Sequence	Used to generate
BTV-8S2F/DNA	5′-GCATTTTCTAGAATGGAGGAGCTAGCGATTCCGATTTAT-3′	pCI-neo BTV-8 Seg-2
BTV-8S2R/DNA	5′-CGTAAAGCGGCCGCGCTATACATTGAGCAGCTTAGTTAACAT-3′	pCI-neo BTV-8 Seg-2
BTV-8S6F/DNA	5′-GCATTTTCTAGAATGGGGAAAATCATAAAGTCC-3′	pCI-neo BTV-8 Seg-6
BTV-8S6R/DNA	5′-AAATGCGCGGCCGCGTCAGGCATTTCTTAAGAAGAG-3′	pCI-neo BTV-8 Seg-6
BTV-8S7F/DNA	5′-GCATTTTCTAGAATGGACACTATCGCTGCAAGAGCA-3′	pCI-neo BTV-8 Seg-7
BTV-8S7R/DNA	5′-CGTAAAGCGGCCGCGCTAAGAGACGTTTGAATGGGTTAC-3′	pCI-neo BTV-8 Seg-7
BTV-8VP2F/VAC	5′-TTTTCCCGGGACCATGGAGGAGCTAGCGATTCCGAT-3′	pSC-11 BTV-8 Seg-2
BTV8VP2R/VAC	5′-TTTTCCCGGGCTATACATTGAGCAGCTTAG-3′	pSC-11 BTV-8 Seg-2
BTV-8VP5F/VAC	5′-TTTTCCCGGGACCATGGGGAAAATCATAAAGTCCCTAAG-3′	pSC-11 BTV-8 Seg-6
BTV-8VP5R/VAC	5′-TTTTCCCGGGTCAGGCATTTCTTAAGAAGAGTGG-3′	pSC-11 BTV-8 Seg-6
BTV-6VP7F/VAC	5′-TTTTCCCGGGACCATGGACACTATCGCTGCAAGAGCAC-3′	pSC-11 BTV-6 Seg-7
BTV-6VP7R/VAC	5′-TTTTCCCGGGCTAAGAGACGTTTGAATGGGTT-3′	pSC-11 BTV-6 Seg-7

XbaI (TCTAGA), SmaI (CCCGGG) and NotI (GCGGCCGC) restriction sequences are underlined.

### Generating Recombinant MVAs

Generation of rMVA BTV-8 VP2, rMVA BTV-8 VP5 and rMVA BTV-6 VP7 was done following standard methods [28]. Seg-7 was cloned from BTV-6 due to problems in cloning Seg-7 from BTV-8. However, it is a highly conserved segment used to identify the two phylogenetic groups [9] and was used in generating rMVA since blast analysis suggests BTV-6 Seg-7 and BTV-8 Seg-7 shares 95% amino acid identity. Briefly, the genes of interest were amplified from pBRT7 BTV-8 Seg-2 NET2006/07, pBRT7 BTV-8 NET2006/07 Seg-6 and pBRT7 BTV-6 NET2006/07 Seg-7 by PCR using gene specific primers (Table 1) containing a SmaI restriction site and cloned into the SmaI site of the standard vaccinia transfer vector pSC-11, downstream of the P7.5 vaccinia promoter, generating plasmids pSC-11 BTV-8 Seg- 2, pSC-11 BTV-8 Seg- 6 and pSC-11 BTV-6 Seg-7, respectively. DF-1 cells infected with MVA at an MOI of 0.1 were transfected with these recombinant plasmids using Lipofectamine™ 2000 Transfection Reagent (Invitrogen), to insert the pSC11 expression cassettes into the thymidine kinase gene locus of the MVA genome by homologous recombination. Recombinant viruses were selected by picking blue plaques following staining with X-gal and amplified in DF-1 cells. Transcription of BTV genes was checked by RT-PCR using specific primers (Table 1) and protein expression checked as detailed below.

### Total RNA Extraction and RT-PCR

CEF were infected with rMVA BTV-8VP2, rMVA BTV-8 VP5 or rMVA BTV-6 VP7. At 24 hours post infection, the infected cells were harvested and centrifuged at 3000 rpm for 5 min. RNA was extracted from the pellet using RNeasy (Qiagen). RT-PCR was performed using Transcriptor One- Step RT-PCR kit (Roche) using primers BTV-8VP2F/VAC, BTV-8VP2R/VAC, BTV-8VP5F/VAC, BTV-8VP5R/VAC, BTV6-VP7F/VAC, and BTV-6VP7R/VAC (Table 1). Wild type MVA was used as negative control.

### Detection of VP2, VP5 and VP7 Expressed by Recombinant MVAs or pCI-neo Plasmids by Indirect Immunofluorescence Assay

Detection of BTV proteins expressed by rMVAs or pCI-neo plasmids was carried out by indirect immunofluorescence assay. CEF cells were grown on cover slips and infected with rMVAs at a MOI of 0.1. On the other hand, Vero cells were transfected with the BTV pCI-neo plasmids using Lipofectamine 2000 (Invitrogen). After 24 h, cells were fixed with 4% paraformaldehyde for 30 min at room temperature. Permeabilization was performed with 0.4% Triton for 15 minutes before incubation with a PBS-20%FBS for 1 hour at room temperature. The cells were reacted with a serum from sheep infected with BTV-8 diluted 1∶800 in PBS-2%FBS for 2 h at 37°C, following washing with PBS. Alexa 488 conjugated polyclonal donkey anti-sheep IgG (Invitrogen) diluted 1∶1000 was used for fluorescent studies. Cells were washed and incubated for 5 minutes with DAPI. Finally cover slips were washed, mounted on glass slides and observed by confocal microscopy.

### Immunisation of IFNAR (−/−) Mice and BTV-8 Challenge

Thirty six female IFNAR (−/−) mice were purchased from B&K Universal Ltd., United Kingdom. All experiments were performed under the guidelines of the European Community (86/609) and were approved by the ethical review committee (reference number: 2008/007) at the Centro de Investigación en Sanidad Animal, INIA, Madrid (CISA). Mice were maintained under pathogen-free conditions and allowed to acclimatize to biosafety level 3 (BSL3) animal facilities at CISA for 1 week before use.

Mice were divided in six groups for the experiment, five groups were immunised (three weeks apart) with the different vaccines while the remaining control group was not vaccinated (Table 2). A suspension of 100 µg of each DNA construct was administered intramuscularly. Doses of 3×10^7^ pfu of rMVA-VP2 or 3×10^5^ pfu of rMVA-VP5 or rMVA-VP7 were inoculated intraperitoneally, since it is the most employed route for MVA inoculation and has the additional advantage of the easier manipulation of animals. The dose used of rMVA-VP5 and rMVA-VP7 was lower because we could not obtain a higher titre of these viruses.

**Table 2:** Vaccination groups, dosage, route and schedule.

Group	Vaccine	Dosage per mouse	Time
1	rMVA-VP2 prime	3×10^7^ pfu	Day 0
1	rMVA-VP2 boost	3×10^7^ pfu	Day 21
2	DNA-VP2 prime	100 ìg	Day 0
2	rMVA-VP2 boost	3×10^7^ pfu	Day 21
3	DNA-VP7 prime	100 ìg	Day 0
3	rMVA-VP7 boost	3×10^5^ pfu	Day 21
4	DNA-VP2, DNA-VP5, DNA-VP7 prime	100 ìg each plasmid	Day 0
4	MVA-VP2, MVA-VP5, MVA-VP7 boost	3×10^7^ pfu, 3×10^5^ pfu, 3×10^5^ pfu each	Day 21
5	MVA-VP2, MVA-VP5, MVA-VP7prime	3×10^7^ pfu, 3×10^5^ pfu, 3×10^5^ pfu each	Day 0
5	MVA-VP2, MVA-VP5, MVA-VP7 boost	3×10^7^ pfu, 3×10^5^ pfu, 3×10^5^ pfu each	Day 21
6	Control	No vaccine	Day 0
6	Control	No vaccine	Day 21

pfu = plaque forming units.

All of the mice were challenged two weeks after the second immunisation, using a lethal dose (10 pfu) of BTV-8 (Belgium/06 isolate) subcutaneously [42]. The clinical signs in vaccinated and control mice were monitored for 13 days post challenge and recorded. Animals that showed severe clinical signs (loss of more than 20% of body weight, frequent hunching, severe conjunctivitis or any other condition that prevented food or water intake) were humanely euthanized.

### Blood Sampling, Virus Detection and Serology

All mice were bled using standard methods [48]. For serological analyses, blood samples were collected on days 0 (before prime immunisation), 20 and 34 post-vaccination and on day 7 and 13 post-challenge. Samples were incubated at room temperature for 30 minutes then centrifuged at 3000 rpm for 10 minutes.

The serum was collected and used for virus neutralisation test (VNT) as described previously [49]. Titres were assigned arithmetically as the dilution of serum that gave a 50% neutralisation endpoint and expressed as log_10_ values.

To determine viraemia titres, a standard plaque assay was conducted on EDTA blood samples collected on days 3, 5, 7, 10 and 12 pc. Whole blood samples were also analysed by a RT-qPCR assay specific for BTV segment 1 as previously described [25].

### Statistical Methods

Differences amongst vaccine groups in outcome following challenge (i.e. survived or dead) were examined using a Fisher exact test. Differences in other measures (clinical score, onset of clinical signs, virus neutralising antibody titres, viral RNA levels and peak viral load) were examined using Kruskal-Wallis tests. If the Kruskal-Wallis test identified significant (P<0.05) differences amongst vaccine groups, these were explored in more detail using Wilcoxon tests for pairwise comparison between groups. Non parametric tests were preferred because of the small group sizes and potential non-normality of the errors.

## Results

### Expression of BTV VP2, VP5 and VP7 from rMVA and DNA Plasmid Vaccines

To test the functionality of the foreign gene expression cassettes in rMVA viruses and pCI-neo plasmids we performed RT-PCR amplification of VP2, VP5 and VP7 from total RNA extracted from rMVA infected or pCIneo transfected cells. We detected VP2, VP5 and VP7 specific cDNA amplicon bands of the expected sizes (∼ 2887, 1580 and 949 nucleotides for VP2, VP5 and VP7 respectively). Standard PCR using HotStart KoD DNA polymerase (Roche) showed no BTV specific DNA bands indicating the amplicons derived from BTV VP2, VP5 or VP7 RNA transcripts and not from plasmid (data not shown).

**Figure 1:**
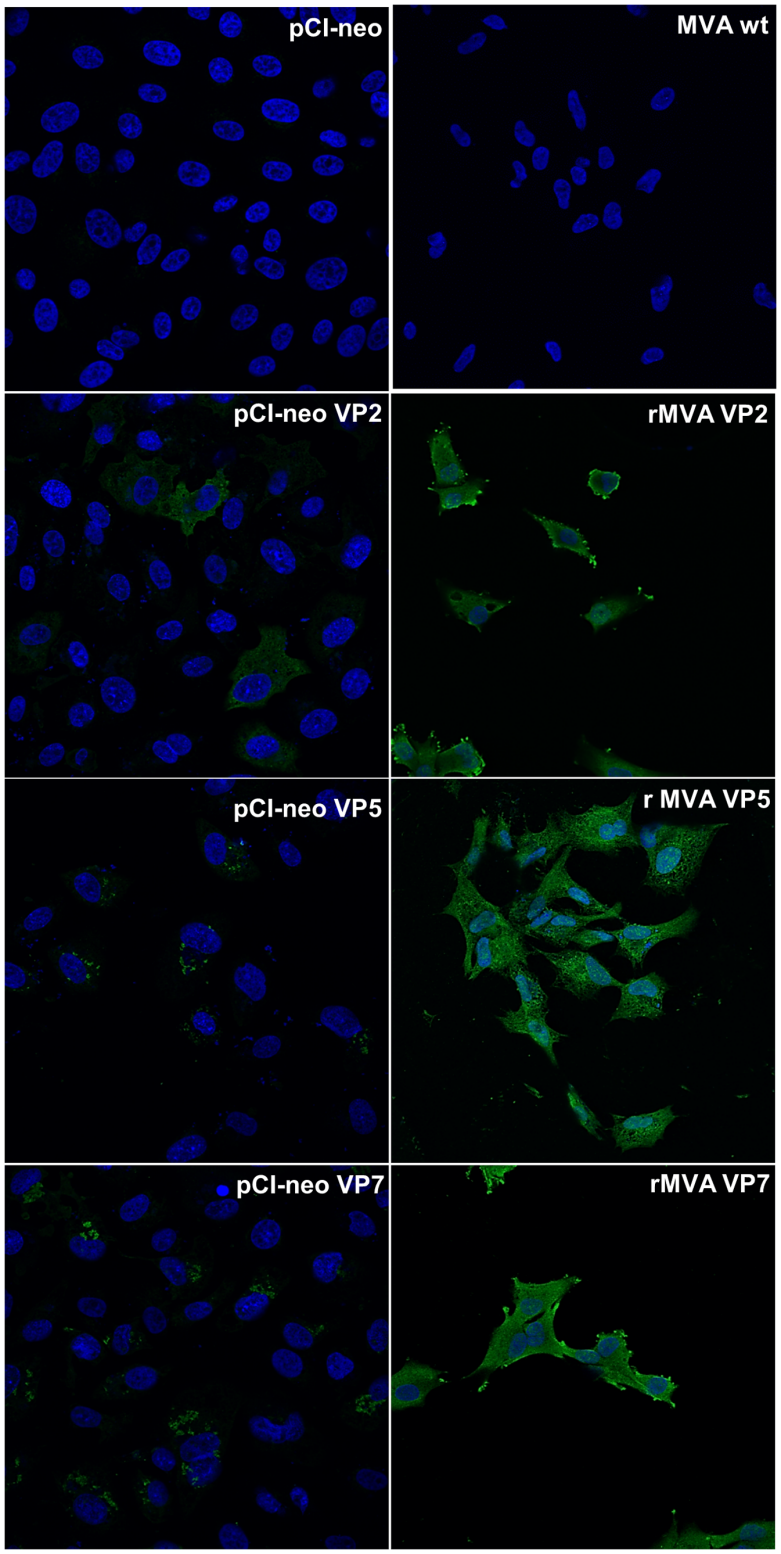
Expression of recombinant BTV proteins from rMVAs and pCi-neo plasmids by immunofluorescence. CEF cells infected with rMVA-VP2, VP5 or VP7, or Vero cells transfected with pCI-neo VP2, VP5 or VP7 were analysis by immunofluorescence assay. Empty MVA and pCI-neo were used as negative controls. Fluorescence was observed on cells using a sheep serum anti BTV-8 followed by Alexa Fluor 488-conjugated donkey anti-sheep IgG. Nuclei were staining with DAPI. BTV protein expression from pCI-neo plasmids or rMVAs encoding VP2, VP5 and VP7 proteins was observed by confocal microscopy.

Moreover, we evaluated the expression of recombinant VP2, VP5 and VP7 by rMVAs or pCI-neo plasmids by immunofluorescence assays using a sheep anti BTV-8 serum (Fig. 1). Expression of these three proteins was confirmed in cells transfected with pCI-neo plasmids or infected with the rMVA viruses used for immunization of IFNAR (−/−) mice.

### Post-challenge Clinical Signs

Mice from groups 1, 2, 4 and 5, vaccinated with VP2 (as sole antigen), or a combination of VP2, VP5 and VP7, using either a homologous (rMVA/rMVA) or a heterologous prime-boost (DNA/rMVA) vaccination regime, showed no clinical signs after challenge with BTV-8 and all of them survived (Table 3). Group 3 (vaccinated with heterologous DNA/rMVA expressing VP7 alone) and group 6 (control) showed severe clinical signs and were euthanized. However, in the VP7 vaccinated mice the onset of clinical signs was significantly delayed (Wilcoxon test: *P* = 0.01) in comparison with the control group. One animal of group 5 died immediately after bleeding without showing clinical signs or viraemia by plaque assay; therefore we considered that the death was not related with the infection.

**Table 3:** Clinical signs after challenge.

Group	Mouse	Vaccine	Clinicalscore	Onset	Survival
1	1.1	MVA/MVA VP2	0		Yes
1	1.2	MVA/MVA VP2	0		Yes
1	1.3	MVA/MVA VP2	0		Yes
1	1.4	MVA/MVA VP2	0		Yes
1	1.5	MVA/MVA VP2	0		Yes
1	1.6	MVA/MVA VP2	0		Yes
2	2.1	DNA/MVA VP2	0		Yes
2	2.2	DNA/MVA VP2	0		Yes
2	2.3	DNA/MVA VP2	0		Yes
2	2.4	DNA/MVA VP2	0		Yes
2	2.5	DNA/MVA VP2	0		Yes
2	2.6	DNA/MVA VP2	0		Yes
3	3.1	DNA/MVA VP7	3	Day 5	No
3	3.2	DNA/MVA VP7	4	Day 5	No
3	3.3	DNA/MVA VP7	4	Day 5	No
3	3.4	DNA/MVA VP7	4	Day 5	No
3	3.5	DNA/MVA VP7	3	Day 6	No
3	3.6	DNA/MVA VP7	6	Day 4	No
4	4.1	DNA/MVA VP2 VP5 VP7	0		Yes
4	4.2	DNA/MVA VP2 VP5 VP7	0		Yes
4	4.3	DNA/MVA VP2 VP5 VP7	0		Yes
4	4.4	DNA/MVA VP2 VP5 VP7	0		Yes
4	4.5	DNA/MVA VP2 VP5 VP7	0		Yes
4	4.6	DNA/MVA VP2 VP5 VP7	0		Yes
5	5.1	MVA/MVA VP2 VP5 VP7	0		Yes
5	5.2	MVA/MVA VP2 VP5 VP7	0		Yes
5	5.3	MVA/MVA VP2 VP5 VP7	0		Yes
5	5.4	MVA/MVA VP2 VP5 VP7	0		Yes
5	5.5	MVA/MVA VP2 VP5 VP7	0		Yes
5	5.6	MVA/MVA VP2 VP5 VP7	0		
6	6.1	Control	4	Day 4	No
6	6.2	Control	4	Day 4	No
6	6.3	Control	6	Day 3	No
6	6.4	Control	5	Day 4	No
6	6.5	Control	4	Day 4	No
6	6.6	Control	3	Day 4	No

Clinical signs from individual mice were recorded and assigned a value according to the following algorithm: reduced activity: 1; frequent hunching: 2; ruffled fur: 1; weight loss: 2; swelling around the eyes: 1. The final clinical score for each animal was the sum of all the values for each individual. Day of onset is the day after challenge when clinical signs appeared.

### Viraemia in Mice after Challenge with BTV-8

**Figure 2:**
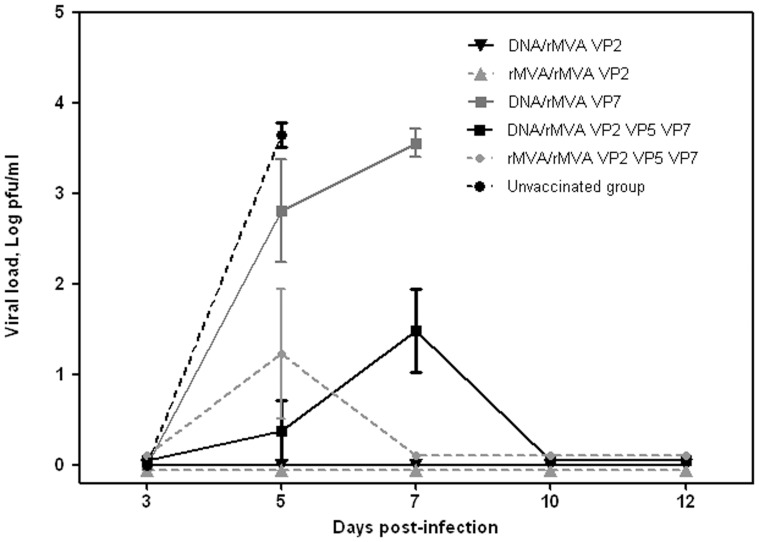
Viral load (log pfu/ml) of blood samples collected from vaccinated and control mice after challenge with BTV-8. Virus was extracted from blood of immunized and non-immunized mice after challenge and determined as described in Materials and Methods. Each point represents the mean values of the viral titer of six animals and standard errors are shown as bars.

No virus was detected in any blood samples taken on day 3 pc. All mice in groups 3 and 6 (vaccinated with VP7 and unvaccinated controls, respectively) developed viraemia titres higher than 1×10^3^ pfu/ml prior to death (Figure 2). Three mice in group 4 and two mice in group 5 (both groups vaccinated with VP2, VP5 and VP7, which all survived) also developed viraemia, albeit at significantly (P = 0,002) lower titres than in groups 3 and 6 (≤3×10^2^ pfu/ml). Although mice in groups 1, 2, 4 and 5 were protected and survived until the end of the experiment, only mice vaccinated with VP2 (Groups 1 and 2) showed no viraemia by plaque assay.

### Detection of Viral RNA in Blood from Mice Post Challenge

The results summarised on Table 4 show that five control mice (Group 6) were positive on day 3 and all six animals were positive on day 5 pc, prior to death. Group 3 (vaccinated with VP7 alone) showed a similar pattern, with three mice becoming positive on day 3. All mice in group 3 were positive on day 5 and the two that survived beyond day 7 were positive on that day. In groups 4 and 5, BTV RNA was detected throughout the experiment, from day 3 to day 12. In Group 1, BTV RNA was detected in five mice but only on day 7 pc; whilst in group 2, BTV RNA was only detected in a single mouse on days 5 and 7 pc. The observed Ct values on day 5 were significantly (P<0.02) higher for mice in groups 1 and 2 compared with those in groups 3, 4, 5 and 6 and for mice in groups 4 and 5 compared with those in group 6. In addition, the observed Ct values on day 7 were significantly (P = 0.05) higher in mice for groups 1 and 2 compared with those in group 4 and 5.

**Table 4:** BTV-8 RNA detection (Ct values) in blood samples collected from vaccinated and control mice after challenge.

Group	Mouse	Vaccine	Day 3	Day 5	Day 7	Day 10	Day 12
1	1.1	MVA/MVA VP2	–	–	34.7	–	–
1	1.2	MVA/MVA VP2	–	–	33.72	–	–
1	1.3	MVA/MVA VP2	–	–	34.24	–	–
1	1.4	MVA/MVA VP2	–	–	–	–	–
1	1.5	MVA/MVA VP2	–	–	33.93	–	–
1	1.6	MVA/MVA VP2	–	–	34.3	–	–
2	2.1	DNA/MVA VP2	–	–	–	–	–
2	2.2	DNA/MVA VP2	–	–	–	–	–
2	2.3	DNA/MVA VP2	–	33.24	32.95	–	–
2	2.4	DNA/MVA VP2	–	–	–	–	–
2	2.5	DNA/MVA VP2	–	–	–	–	–
2	2.6	DNA/MVA VP2	–	–	–	–	–
3	3.1	DNA/MVA VP7	35.15	26.34	†		
3	3.2	DNA/MVA VP7	33.86	24.67	†		
3	3.3	DNA/MVA VP7	–	30.67	†		
3	3.4	DNA/MVA VP7	–	32.33	25.6	†	
3	3.5	DNA/MVA VP7	–	32.7	31.2	†	
3	3.6	DNA/MVA VP7	31.79	23.85	†		
4	4.1	DNA/MVA VP2 VP5 VP7	–	34.9	31.55	31.37	32.8
4	4.2	DNA/MVA VP2 VP5 VP7	40.35	32.51	30.37	34.62	33.64
4	4.3	DNA/MVA VP2 VP5 VP7	33.91	31.47	29.96	–	–
4	4.4	DNA/MVA VP2 VP5 VP7	34.2	31.55	30.7	31.97	–
4	4.5	DNA/MVA VP2 VP5 VP7	31.9	31.47	31.9	31.33	33.62
4	4.6	DNA/MVA VP2 VP5 VP7	–	33.65	35.07	–	34.2
5	5.1	MVA/MVA VP2 VP5 VP7	–	30.42	31.16	–	–
5	5.2	MVA/MVA VP2 VP5 VP7	–	31.1	31.32	32.57	33.03
5	5.3	MVA/MVA VP2 VP5 VP7	–	35.04	33.84	32.44	–
5	5.4	MVA/MVA VP2 VP5 VP7	34.13	42.06	32.61	30.85	34.77
5	5.5	MVA/MVA VP2 VP5 VP7	33.85	–	34.49	–	–
5	5.6	MVA/MVA VP2 VP5 VP7	34.39	30.47	†	†	
6	6.1	Control	30.34	25.94	†		
6	6.2	Control	32.4	24.82	†		
6	6.3	Control	31.38	24.07	†		
6	6.4	Control	34.56	28.02	†		
6	6.5	Control	–	29.09	†		
6	6.6	Control	33.87	26.46	†		

No Ct value for a particular sample is indicated by –.

Sample not taken due to previous death of the animal is indicated by †.

### Neutralising Antibodies in Vaccinated Mice

**Figure 3:**
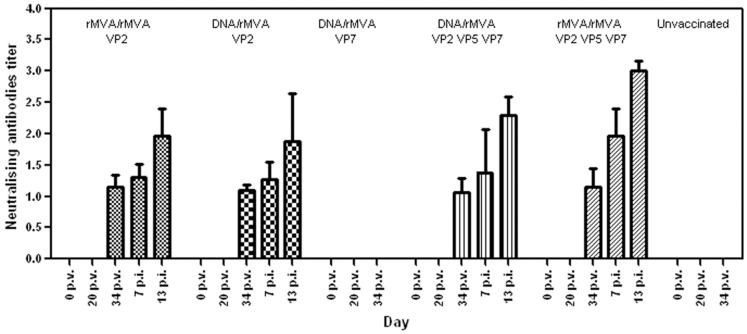
Virus neutralising antibodies in serum of mice following vaccination and challenge. Mean values of AHSV-4 neutralising antibodies measured in mice groups at different times after vaccination and after challenge. Titres are assigned arithmetically as the dilution of serum that will give a 50% neutralisation endpoint and expressed as log10 values. Standard deviations are shown as error bars. p.v. = post-vaccination, p.i. = post-infection.

Virus neutralising antibodies against BTV-8 were detected on day 34 (two weeks post boost) in all mice that had received VP2 based vaccines (DNA/rMVA or rMVA/rMVA), either alone or in combination with other BTV proteins (Groups 1, 2, 4, and 5) (Figure 3). Titres ranged between 1.06 and 1.15 (log_10_ VNT) and did not differ significantly (P>0.05) amongst these groups. Titres rose after challenge ranged from 1.88 to 3. No neutralising antibodies were detected in serum from mice vaccinated with VP7 alone, or in serum from the control group (Group 6). The level of neutralising antibodies post-challenge was significantly (P<0.02) higher in group 5 compared with groups 1, 2 and 4 possibly caused by the partial replication of the challenge viruses. In contrast, groups 1, 2 and 4, which developed slightly higher neutralising antibodies post vaccination, were more effectively protected, with lower levels of challenge-virus replication, and developed lower neutralising antibody levels by day 13 pc.

## Discussion

Classical vaccines have been used against BTV in the field, however recombinant vaccines offer good immunogenicity and are safer avoiding reversion to virulence (e.g. by reassortment with wild type strains), contamination with toxic compounds used for inactivation and any risk of incomplete inactivation of whole-cell vaccines. Moreover, recombinant marker vaccines allow the distinction between vaccinated and naturally infected animals (DIVA). Recombinant live viruses and DNA plasmids have been widely used as delivery systems for expression of foreign antigens and as vaccine candidates for infectious diseases and cancer [50], [51], [52], [53]. DNA vaccines appear to be more efficacious when they are used to prime immune responses in heterologous vaccination regimes with recombinant Adenovirus or Poxvirus vectors. Indeed, it has been described that these heterologous protocols induced higher frequencies of CTLs than when each immunogen was administered separately [54], [55]. However, homologous prime-boost vaccination with recombinant MVA virus (Family *Poxviridae*) has been used in many successful vaccination studies against hepatitis C [56], HIV [57] and influenza A/H5N1 [58] viruses, demonstrating its efficacy as potential vaccine following that regime. Indeed, it has been described that recombinant MVA vaccines can be administered repeatedly without interference of vector-specific antibodies induced after the first immunization and without loss of booster antibody responses against the target antigen after subsequent immunization [59], [60].

In the case of BTV, previous results of DNA/rMVA prime-boost vaccination using VP2, VP5 and VP7 from BTV-4 in a mouse model [29] demonstrated the efficacy of this approach. On the other hand, in earlier studies with AHSV [41], [61] we also observed that rMVA expressing AHSV-4 VP2, when used in a homologous prime-boost vaccination regime, induced good neutralizing antibody responses in ponies and neutralizing antibodies and protection in IFNAR (−/−) mice. In this study, we have compared directly the efficacy of homologous rMVA/rMVA and heterologous DNA/rMVA prime-boost vaccination regimes expressing either VP2 alone, VP7 alone or a combination of VP2, VP5 and VP7 proteins in IFNAR (−/−) mice.

Our results showed that both homologous (rMVA/rMVA) or heterologous (DNA/rMVA) prime boost vaccinations, expressing either BTV-8 VP2 alone or a combination of three major BTV proteins VP2, VP5 and VP7, induced protective immunity against BTV-8 challenge in mice. We showed very similar levels of efficacy of both vaccination regimes. The two groups of mice vaccinated with BTV-8 VP2 alone, using either a heterologous DNA/rMVA or homologous rMVA/rMVA strategy, were completely protected against clinical signs of BTV infection and had no detectable viraemia by plaque assay. Only low level of BTV RNA was detected in some individuals by qRT-PCR.

The present study also showed that the addition of DNAs or MVAs expressing VP5 and VP7 was not critical for the induction of neutralising antibodies and protection. These vaccines did not improve the protection induced by BTV-8 VP2 alone following DNA/rMVA or rMVA/rMVA vaccinations, since vaccination with BTV-8 VP2 alone was enough to protect animals. Indeed, mice immunized with a combination of recombinant vaccines each expressing VP2, VP5 or VP7 showed higher levels of BTV virus and BTV RNA in blood than mice immunised with VP2 alone. This result contrasts with those observed in previous studies with MVA BTV-4 [28], where VP2, VP5 and VP7 were necessary to confer protective immunity in IFNAR (−/−) mice. In our present study the protein VP2 was expressed from BTV-8, indicating that there could be differences in immunogenicity between same proteins of different serotypes. Moreover, VP2 isolated from BTV virus particles or as expressed by recombinant baculoviruses, has previously been used to protect sheep from BTV challenge [13], [38], [62]. In other studies BTV-8 VP2 alone was not enough to confer protection against challenge in mice, nevertheless the viral vectors used in those studies were different from MVA [49], [63]. At present it is not clear why in some circumstances VP2 alone is enough to induce protective immunity. Further studies would be necessary to better characterise the induction of immune responses following these vaccinations.

Although VP7 does not raise antibodies that can neutralise intact BTV particles, it can provide partial protection via a cell mediated immune response, and its incorporation is thought to enhance the efficacy of VP2 and VP5 vaccines [38], [64]. In the current study, although vaccination with VP7 alone did not protect IFNAR (−/−) mice against BTV-8 challenge, animals showed a delayed onset of clinical signs and the survival time was slightly longer than in the non-vaccinated mice.

In summary, our results show that VP2 expressed in vivo using a heterologous or homologous prime boost vaccination (DNA/rMVA or rMVA/rMVA), can generate immunity against BTV-8 in IFNAR (−/−) mice, protecting them against a lethal challenge, and that a homologous vaccination regime using rMVA was at least as effective as a DNA/rMVA heterologous approach. However, further work will be needed to test and validate the use and efficacy of these BTV-subunit vaccine candidates in ruminants, the natural hosts for BTV infection.
